# Prognostic Significance of COVID-19 Receptor ACE2 and Recommendation for Antihypertensive Drug in Renal Cell Carcinoma

**DOI:** 10.1155/2020/2054376

**Published:** 2020-11-25

**Authors:** Kihun Kim, Yeji Ko, Dai Sik Ko, Yun Hak Kim

**Affiliations:** ^1^Department of Occupational and Environmental Medicine, Kosin University Gospel Hospital, Republic of Korea; ^2^Department of Statistics, University of Michigan, Michigan, USA; ^3^Division of Vascular Surgery, Department of Surgery, Gachon University Gil Medical Center, Republic of Korea; ^4^Department of Biomedical Informatics, School of Medicine, Pusan National University, Republic of Korea; ^5^Department of Anatomy, School of Medicine, Pusan National University, Republic of Korea

## Abstract

**Purpose:**

Owing to its worldwide spread, the coronavirus disease (COVID-19) epidemic was declared a pandemic by the World Health Organization on March 11, 2020. Angiotensin-converting enzyme 2 (ACE2) is the outer surface protein of the cell membrane that is abundantly distributed in the heart, lungs, and kidneys and plays an important role in molecular docking of the severe acute respiratory syndrome coronavirus 2. In this study, we aimed to analyze the difference in the survival rate according to ACE2 expressions in pan-cancer.

**Materials and Methods:**

We downloaded clinical and genomic data from The Cancer Genome Atlas. We used Kaplan-Meier with a log-rank test, and the Cox proportional hazards regression to analyze prognostic significance.

**Results:**

In the Kaplan-Meier curve, clear cell renal cell carcinoma (ccRCC), uveal melanoma, and prostate adenocarcinoma showed statistical significance. In the Cox regression, thyroid carcinoma and glioblastoma multiforme and ccRCC showed significant results. Only ccRCC had statistical significance, and high ACE2 expression is related to good prognosis. It is known that the ACE inhibitor, a primary antihypertensive agent, increases ACE2 expression.

**Conclusion:**

Based on these results, we believe that the ACE inhibitor will be important to increase the lifespan of ccRCC patients. This study is the first research to offer a recommendation on the use of anti-hypertensive drugs to ccRCC patients.

## 1. Introduction

Coronavirus (CoV) belongs to a family of viruses characterized by highly diverse, enveloped, positive-sense, and single-stranded RNA genomes [1]. They cause respiratory, gastrointestinal, hepatic, and neurological symptoms in animals or humans according to their type [2]. The well-known Middle East respiratory syndrome CoV and severe acute respiratory syndrome CoV (SARS-CoV) are fatal to humans, while human CoV (HCoV) OC43, HCoV-229E, and HCoV-NL63 cause only mild respiratory symptoms [1, 3–5]. In December 2019, cases of unknown pneumonia occurred in Wuhan, Hubei Province, China. Subsequently, the causative virus was extracted from human patients, and a molecular analysis revealed that it was a novel coronavirus [6]. The virus was tentatively named “severe acute respiratory syndrome coronavirus 2 (SARS-CoV-2)” by the International Committee on Taxonomy of Viruses [7]. The World Health Organization (WHO) named the disease caused by SARS-CoV-2 as COVID-19 on February 11, 2020 [8]. With its worldwide spread, the COVID-19 epidemic was declared a pandemic by the WHO on March 11, 2020 [9].

Angiotensin-converting enzyme 2 (ACE2) is the outer surface protein of the cell membrane that is abundantly distributed in the heart, lungs, and kidneys [10–12]. ACE2 is a functional receptor of SARS-CoV [13, 14]. SARS-CoV-2 shares 80% similarity with the genome of SARS-CoV, and its cell entry mechanism is mediated by the ACE2 receptor [13, 15]. ACE2 is currently emerging as a new research topic in the wake of SARS-CoV infection. The ACE inhibitor, which is widely used as a therapeutic agent for hypertension, is reported to upregulate the ACE2 receptor expression [16]. The use of ACE inhibitors has been suggested to increase the susceptibility to COVID-19 and worsen the COVID-19 outcome through an increase in the viral load [17]. However, owing to the insufficient evidence at present, the management of hypertension in patients with COVID-19 is controversial [18].

TCGA is a large prospective cohort with data on several variables (demographic, clinical, and genomic data) of approximately 11,000 patients for 33 common cancers [19]. Especially in the field of big data, high-dimensional genomics is available [20]. As the importance of managing cancer diseases increases in the COVID-19 pandemic era, we analyzed the difference in the survival rate according to ACE2 expression levels in 31 cancers by using The Cancer Genome Atlas (TCGA) dataset. Accordingly, we aimed to provide recommendations for the treatment of viral infection or use of ACE inhibitors in certain patients with cancer.

## 2. Material and Methods

### 2.1. Patients

The clinical and genomic data of 33 cancers listed in TCGA were downloaded from the Firehose database (https://gdac.broadinstitute.org/) in February 2020. All TCGA data were available without restrictions from publications or presentations in accordance with TCGA publication guidelines. Patients' clinical variables such as cancer stage, age, sex, and censoring status, as well as ACE2 expression levels, were also extracted. Patient data with insufficient clinical or genetic information were excluded. Two cancers without ACE2 expression levels were excluded from the analysis.

### 2.2. Statistical Analyses

**Figure 1:**
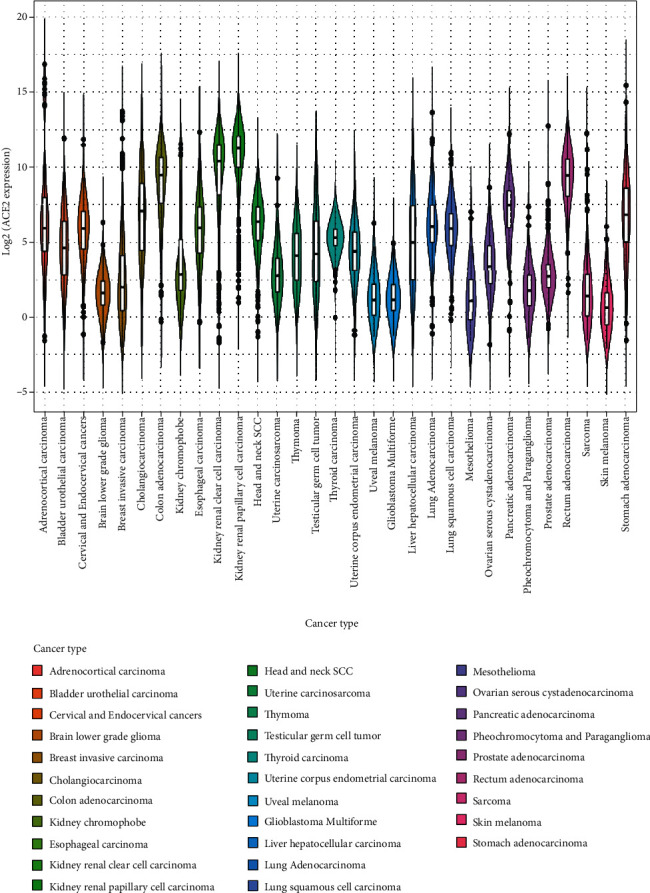
Violin plot of the ACE2 expression levels (log2 transformation).

A violin plot with log2 transformed ACE2 expression on the *y*-axis and cancer types on the *x*-axis was created to compare the gene expression levels between the different cancers (Figure 1). In the present study, we performed a Kaplan-Meier analysis and log-rank tests. A continuous ACE2 expression level was converted to a binary factor low (0) and high (1), with a median cutoff; two distinct survival distributions expressed in binary form were developed. Cancers with positive and negative relationships with the ACE2 expression level and survival rate were defined as “positive” and “negative” cancers, respectively. Nineteen cancers were positive, and 12 were negative on the basis of the odds ratios.

**Figure 2:**
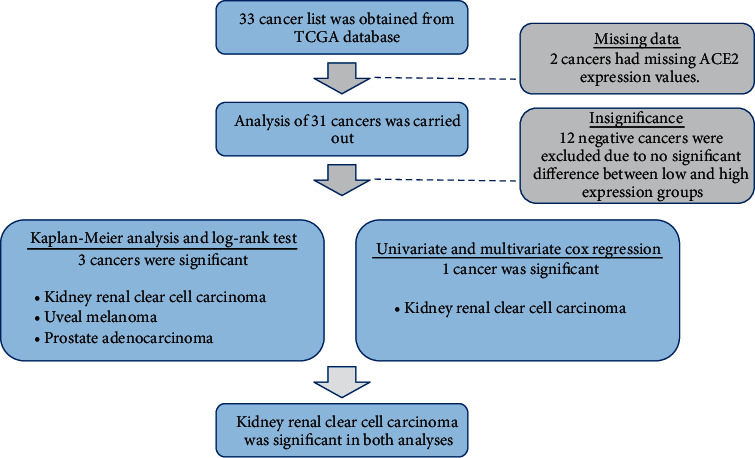
Flowchart for statistical analysis.

We also used univariable and multivariable Cox proportional hazards regression models for all 31 cancers to estimate the hazard ratio, 95% confidence interval, and *p* value of ACE2 and the other clinical variables in the data for each cancer as we described previously [20, 21]. Finally, we checked the significance of the values and combined the results with the survival analysis output to conclude which type of cancer could be most affected by the ACE2 gene. The concordance index was used to evaluate the prediction accuracy of our statistical models. A flowchart is provided in Figure 2 to visually represent the process. All statistical analyses were performed using R version 3.6.0 (R Foundation for Statistical Computing, Vienna, Austria).

## 3. Results

### 3.1. Patient Data and ACE2 Expression

The numbers of samples obtained from TCGA dataset for all cancer types are shown in Table 1. Demographic and clinical details are not shown in this paper. Two cancers, clear cell renal cell carcinoma and renal papillary cell carcinoma, stood out in the violin plot (Figure 1) because their median log2 transformed ACE2 expression levels were >10. Eight cancers, namely, brain lower-grade glioma, invasive breast carcinoma, uveal melanoma, glioblastoma multiforme, mesothelioma, pheochromocytoma and paraganglioma, sarcoma, and skin melanoma, were found to have median values of <2.5.

### 3.2. Survival Analyses

**Figure 3:**
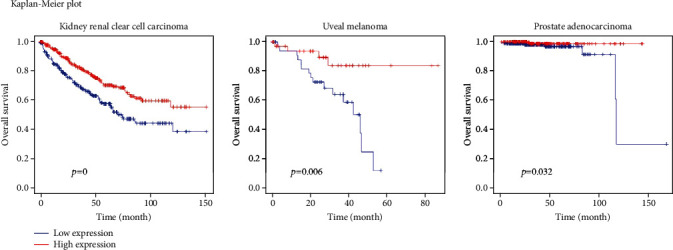
Survival plots of patients with cancers who had significant log-rank test results.

None of the negative cancers had significant log-rank test results. Of the 19 positive cancers, three, namely clear cell renal cell carcinoma, uveal melanoma, and prostate adenocarcinoma, had *p* values of <0.05 and thus were considered significant in the Kaplan-Meier analysis and log-rank test (Figure 2). The survival plots of these cancers are shown in Figure 3.

Although two negative cancers, thyroid carcinoma and glioblastoma multiforme, and two positive cancers, adrenocortical carcinoma and mesothelioma, were significant in the multivariable regression results, they were still insignificant in the univariable Cox regression models (Figure 2). As the multivariable models of these cancers could elicit a false idea of interaction effects between covariates, we decided not to further investigate them.

**Figure 4:**
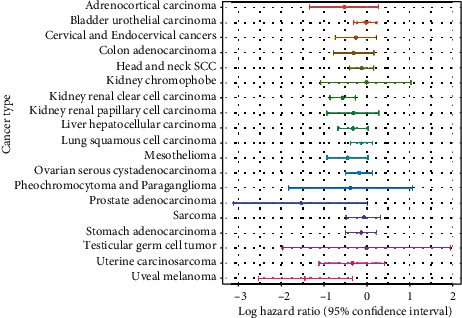
Forest plot of the hazard ratios (univariable) of 31 cancers.

**Figure 5:**
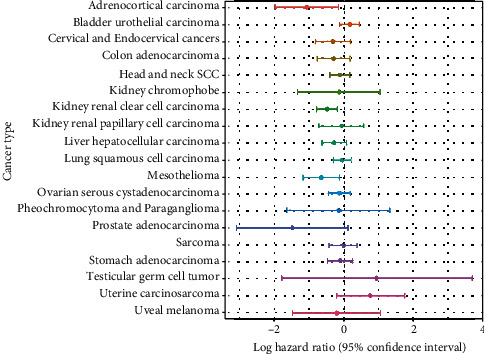
Forest plot of the hazard ratios (multivariable) of 31 cancers.

Clear cell renal cell carcinoma was the only cancer with significant results in both the univariable and multivariable Cox regression analyses (Figure 2). The cancer had hazard ratios of 0.571884 and 0.61273 in the univariable and multivariable Cox regression models, respectively. These values indicate that the relative risks of death in the defined period were approximately 43% and 39% lower, respectively, in the high gene expression group than in the low gene expression group. These results were also observed in a forest plot, as only clear cell renal cell carcinoma had a confidence interval of <0 in both the univariable and multivariable regression analyses (Figures 4 and 5).

The concordance indexes of the univariable Cox regression models for clear cell renal cell carcinoma, uveal melanoma, and prostate adenocarcinoma were 0.6874, 0.59, and 0.5351, respectively (Table 2). As a model with a concordance index of >0.55 is considered a good model, this implies a good predictive ability for clear cell renal cell carcinoma and uveal melanoma [22]. We conclude that high ACE2 gene expression levels could positively influence the survival rate of patients with clear cell renal cell carcinoma and have high model accuracy levels.

## 4. Discussion

In this study, we analyzed the survival rate according to ACE2 expression level for various cancers by using statistical models such as the Kaplan-Meier analysis and log-rank test and univariable and multivariable Cox regression analyses. In addition, the survival prediction performance of ACE2 expression level was evaluated with the concordance index to corroborate the significantly different results from the previous tests.

The results of the Kaplan-Meier analysis and log-rank test indicated significant differences in survival rates among patients with renal cell carcinoma, uveal melanoma, and prostate adenocarcinoma according to ACE2 expression level. The results of the univariable and multivariable Cox regression analyses indicated that the ACE2 expression level had a significant effect on the survival rates of patients with renal clear cell carcinoma, thyroid carcinoma, and glioblastoma multiforme. Only clear cell renal cell carcinoma showed significant results in both the Kaplan-Meier analysis and log-rank test/univariable and multivariable Cox regression analyses. Therefore, we could assume that in clear cell renal cell carcinoma, the ACE2 expression level is strongly associated with survival rate and acts as an important causal factor for predicting the survival rate. The concordance index of clear cell renal cell carcinoma was 0.687, confirming its good survival prediction performance. As the hazard ratio of clear cell renal cell carcinoma was significantly <1 in both the univariable and multivariable Cox regression analyses, we estimated that the higher the patient's ACE2 expression level, the lower the patient's estimated risk, which corresponds to a higher survival rate.

The ACE inhibitor is used as an initial therapy for high blood pressure in many situations (e.g., heart failure with reduced ejection fraction and chronic kidney disease) [23–26]. It inhibits the conversion of angiotensin I to angiotensin II to reduce the activity of the renin-angiotensin-aldosterone system [27, 28]. The use of ACE inhibitor has been shown to increase the ACE2 expression level in human and animal studies [16, 29]. Therefore, we assumed that the use of ACE inhibitors for controlling high blood pressure in patients with clear cell renal cell carcinoma will increase their ACE2 expression levels, which may help to improve survival rates if the administration of the ACE inhibitor is prioritized when choosing a high blood pressure control drug for these patients.

As mentioned earlier, ACE2, which is known to play an important role in molecular docking in the cell entry process of CoVs, is associated with mainly respiratory symptoms [2, 30]. In addition, an increased ACE2 expression level induces more CoV viral loads, including those of SARS-CoV and SARS-CoV-2. Cancers with significant results in this study were less relevant to respiratory viruses. Therefore, ACE2 expression level and survival rate seem to have no significant correlation in patients with respiratory cancer. The cancers with significant results in this study are presumed to be less relevant to respiratory viruses, including CoVs. Therefore, we speculated that SARS-CoV infection does not have a significant effect on the survival rate of certain cancer patients.

In several carcinomas (breast cancer, uterus corpus endometrial carcinoma, kidney renal papillary carcinoma, non-small-cell lung cancer, hepatocellular carcinoma, and pancreatic cancer), ACE2 expression was downregulated, which is thought to be a poor prognostic factor [31–35]. This is consistent with the content of our study. In a recent paper, CD4 memory, CD8 effector, T helper cell, dendritic cell, and NK cell, which are associated with tumor infiltration, are reported to increase in ccRCC tissues [31]. The decrease in ACE2 expression is related to tumor proliferation, stemness, and epithelial-mesenchymal transition even at the micro environmental level [36]. It was assumed that the change in the immune microenvironment resulted in a difference in survival rates, and the increase in ACE2 expression is thought to mainly play an antitumor role. However, to the author's knowledge, it is assumed that the clear mechanisms of ACE2 expression and ccRCC are not established. The further clinical validation study that correlated between ACE2 expression and survival rates is required.

The limitation of this study is that the statistical analyses were conducted only with a single cohort. The results must be verified through a multicohort analysis. Second, the suggested drugs for hypertension and viral infections were hypothesized through statistical results, and the actual clinical outcomes require validation through further clinical trials. Third, because the number of samples collected for each cancer varied (range, 68–985), the power of the statistical results also varied among the cancer types.

## 5. Conclusion

**Figure 6:**
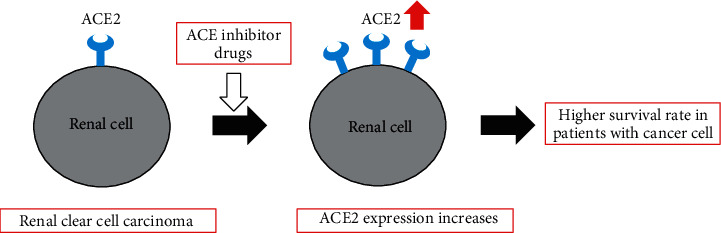
Graphical summary of this study.

By using big data, we analyzed the differences in survival rates among certain cancers according to ACE2 expression, which is known to be important for the entry of SARS-CoV2, the virus responsible for the COVID-19 pandemic. The ACE2 expression level was highly relevant to the survival rate of patients with clear cell renal cell carcinoma and could be an important factor for predicting survival. The results of this study might be useful for further studying ACE-related treatments, care, and outcomes in patients with ccRCC (Figure 6).

## Figures and Tables

**Table 1:** Number of samples.

Cancer type	Number (*n*)	Cancer type	Number (*n*)
Adrenocortical carcinoma	68	Uterine corpus endometrial carcinoma	144
Bladder urothelial carcinoma	425	Uveal melanoma	74
Cervical and endocervical cancers	276	Glioblastoma multiforme	143
Brain lower-grade glioma	418	Liver hepatocellular carcinoma	359
Breast invasive carcinoma	985	Lung adenocarcinoma	367
Cholangiocarcinoma	36	Lung squamous cell carcinoma	539
Colon adenocarcinoma	313	Mesothelioma	86
Kidney chromophobe	89	Ovarian serous cystadenocarcinoma	277
Esophageal carcinoma	172	Pancreatic adenocarcinoma	163
Clear cell renal cell carcinoma	583	Pheochromocytoma and paraganglioma	187
Renal papillary cell carcinoma	282	Prostate adenocarcinoma	494
Head and neck squamous cell carcinoma	463	Rectal adenocarcinoma	82
Uterine carcinosarcoma	46	Sarcoma	253
Thymoma	117	Skin melanoma	421
Testicular germ cell tumor	133	Stomach adenocarcinoma	329
Thyroid carcinoma	488		

**Table 2:** Concordance index scores.

Cancer type	Concordance index score
Renal cell clear cell carcinoma	0.687
Uveal melanoma	0.590
Prostate adenocarcinoma	0.535

## Data Availability

Data are available upon TCGA publication guideline.

## References

[1] Zumla A., Chan J. F., Azhar E. I., Hui D. S., Yuen K.-Y. (2016). Coronaviruses -- drug discovery and therapeutic options. *Nature Reviews Drug Discovery*.

[2] He F., Deng Y., Li W. (2020). Coronavirus disease 2019: what we know?. *Journal of Medical Virology*.

[3] Channappanavar R., Zhao J., Perlman S. (2014). T cell-mediated immune response to respiratory coronaviruses. *Immunologic Research*.

[4] Cheng V. C. C., Lau S. K. P., Woo P. C. Y., Yuen K. Y. (2007). Severe acute respiratory syndrome coronavirus as an agent of emerging and reemerging infection. *Clinical Microbiology Reviews*.

[5] Chan J. F. W., Lau S. K. P., To K. K. W., Cheng V. C. C., Woo P. C. Y., Yuen K. Y. (2015). Middle East respiratory syndrome coronavirus: another zoonotic betacoronavirus causing SARS-like disease. *Clinical Microbiology Reviews*.

[6] Sun J., He W. T., Wang L. (2020). COVID-19: epidemiology, evolution, and cross-disciplinary perspectives. *Trends in Molecular Medicine*.

[7] Gorbalenya A. E. (2020). *Severe Acute Respiratory Syndrome-Related Coronavirus–The Species and its Viruses, a Statement of the Coronavirus Study Group*.

[8] Zhao S., Lin Q., Ran J. (2020). Preliminary estimation of the basic reproduction number of novel coronavirus (2019-nCoV) in China, from 2019 to 2020: a data-driven analysis in the early phase of the outbreak. *International Journal of Infectious Diseases*.

[9] Anjorin A. A. A. (2020). The coronavirus disease 2019 (COVID-19) pandemic: a review and an update on cases in Africa. *Asian Pacific Journal of Tropical Medicine*.

[10] Hamming I., Timens W., Bulthuis M., Lely A., Navis G., van Goor H. (2004). Tissue distribution of ACE2 protein, the functional receptor for SARS coronavirus. A first step in understanding SARS pathogenesis. *The Journal of Pathology*.

[11] Donoghue M., Hsieh F., Baronas E. (2000). A novel angiotensin-converting enzyme–related carboxypeptidase (ACE2) converts angiotensin I to angiotensin 1-9. *Circulation Research*.

[12] Baraniuk C. (2020). *Receptor for SARS-CoV-2 Present in Wide Variety of Human Cells*.

[13] Yan R., Zhang Y., Li Y., Xia L., Guo Y., Zhou Q. (2020). Structural basis for the recognition of SARS-CoV-2 by full-length human ACE2. *Science*.

[14] Guo J., Wei X., Li Q. (2020). Single-cell RNA analysis on ACE2 expression provides insights into SARS-CoV-2 potential entry into the bloodstream and heart injury. *Journal of Cellular Physiology*.

[15] Li W., Moore M. J., Vasilieva N. (2003). Angiotensin-converting enzyme 2 is a functional receptor for the SARS coronavirus. *Nature*.

[16] Ferrario C. M., Jessup J., Chappell M. C. (2005). Effect of angiotensin-converting enzyme inhibition and angiotensin II receptor blockers on cardiac angiotensin-converting enzyme 2. *Circulation*.

[17] Kuster G. M., Pfister O., Burkard T. (2020). SARS-CoV2: should inhibitors of the renin–angiotensin system be withdrawn in patients with COVID-19?. *European Heart Journal*.

[18] Patel A. B., Verma A. (2020). COVID-19 and angiotensin-converting enzyme inhibitors and angiotensin receptor blockers: what is the evidence?. *JAMA*.

[19] Liu J., Lichtenberg T., Hoadley K. A. (2018). An integrated TCGA pan-cancer clinical data resource to drive high-quality survival outcome analytics. *Cell*.

[20] Pak K., Oh S. O., Goh T. S. (2020). A user-friendly, web-based integrative tool (ESurv) for survival analysis: development and validation study. *Journal of Medical Internet Research*.

[21] Goh T. S., Lee J. S., Il Kim J. (2018). Prognostic scoring system for osteosarcoma using network-regularized high-dimensional Cox-regression analysis and potential therapeutic targets. *Journal of Cellular Physiology*.

[22] Harrell F. E., Lee K. L., Mark D. B. (1996). Multivariable prognostic models: issues in developing models, evaluating assumptions and adequacy, and measuring and reducing errors. *Statistics in Medicine*.

[23] Chobanian A. V., Bakris G. L., Black H. R. (2003). The seventh report of the joint national committee on prevention, detection, evaluation, and treatment of high blood pressure: the JNC 7 report. *JAMA*.

[24] Mancia G., Fagard R., Narkiewicz K. (2013). 2013 practice guidelines for the management of arterial hypertension of the European Society of Hypertension (ESH) and the European Society of Cardiology (ESC). *Journal of Hypertension*.

[25] Rosendorff C., Black H. R., Cannon C. P. (2007). Treatment of hypertension in the prevention and management of ischemic heart disease: a scientific statement from the American Heart Association Council for High Blood Pressure Research and the Councils on Clinical Cardiology and Epidemiology and Prevention. *Circulation*.

[26] Williams B., Mancia G., Spiering W. (2018). 2018 ESC/ESH guidelines for the management of arterial hypertension. *European Heart Journal*.

[27] Jandeleit-Dahm K., Cooper M. E. (2006). Hypertension and diabetes: role of the renin-angiotensin system. *Endocrinology and Metabolism Clinics*.

[28] Wang W., McKinnie S. M. K., Farhan M. (2016). Angiotensin-converting enzyme 2 metabolizes and partially inactivates pyr-apelin-13 and apelin-17: physiological effects in the cardiovascular system. *Hypertension*.

[29] Furuhashi M., Moniwa N., Mita T. (2015). Urinary angiotensin-converting enzyme 2 in hypertensive patients may be increased by olmesartan, an angiotensin II receptor blocker. *American Journal of Hypertension*.

[30] Zhang Y., Zheng N., Hao P., Cao Y., Zhong Y. (2005). A molecular docking model of SARS-CoV S1 protein in complex with its receptor, human ACE2. *Computational Biology and Chemistry*.

[31] Yang W., Li L., Zhang K. (2021). ACE2 correlated with immune infiltration serves as a novel prognostic biomarker in clear cell renal cell carcinoma: implication for COVID-19. *International Journal of Biological Sciences*.

[32] Zhang Q., Lu S., Li T. (2019). ACE2 inhibits breast cancer angiogenesis via suppressing the VEGFa/VEGFR2/ERK pathway. *Journal of Experimental & Clinical Cancer Research*.

[33] Feng Y., Wan H., Liu J. (2010). The angiotensin-converting enzyme 2 in tumor growth and tumor-associated angiogenesis in non-small cell lung cancer. *Oncology Reports*.

[34] Zhou L., Zhang R., Yao W. (2009). Decreased expression of angiotensin-converting enzyme 2 in pancreatic ductal adenocarcinoma is associated with tumor progression. *The Tohoku Journal of Experimental Medicine*.

[35] Ye G., Qin Y., Lu X. (2015). The association of renin-angiotensin system genes with the progression of hepatocellular carcinoma. *Biochemical and Biophysical Research Communications*.

[36] Zhang Z., Li L., Li M., Wang X. (2020). The SARS-CoV-2 host cell receptor ACE2 correlates positively with immunotherapy response and is a potential protective factor for cancer progression. *Computational and Structural Biotechnology Journal*.

